# Assessing the Relative Integrity of Formed Cardiac Linear Lesions by Recording Both Focal Monophasic Action Potentials and Contact Forces: A Technical Brief

**DOI:** 10.1109/JTEHM.2015.2473856

**Published:** 2015-08-27

**Authors:** Mark A. Benscoter, Paul A. Iaizzo

**Affiliations:** Biomedical Engineering DepartmentUniversity of MinnesotaMinneapolisMN55455USA; Department of EngineeringMayo ClinicRochesterMN55902USA; Department of SurgeryUniversity of MinnesotaMinneapolisMN55455USA

**Keywords:** Atrial fibrillation, atrial flutter, monophasic action potential, catheter ablation, coronarysinus, mitral isthmus

## Abstract

The use of therapeutic ablation in patients with atrial fibrillation has become a mainstay in the treatment of this disease, yet often these individuals require multiple procedures. In other words, successful first time treatments are impacted by challenges, including the generation of linear lesions in certain anatomies like the mitral isthmus of the left atrium. Hence, there is a need to find ways to address the presence of unwanted conduction gaps at the time of lesion creation. In this paper, we describe a novel approach to examine conduction gaps, by using a proof of concept device to examine local electrical activation within the cardiac areas of an applied lesion, i.e., to locate gaps in the lesion set. To accomplish this, both epicardial and endocardial linear ablation lines composed of spot lesions with conduction gaps were created in a porcine model. The forces necessary to elicit monophasic action potentials (MAP) were collected from >200 measurements on the epicardium of the right ventricle. Ablations were then performed on the ventricular epicardium and left atrial mitral isthmus endocardially, while recording MAPs. We were able to successfully demonstrate the use of a proof of concept device to identify conduction gaps in linear lesion sets; furthermore, we were able to determine required contact forces to appropriately determine focal electrical changes of the underlying tissues. New catheter designs that incorporate capabilities to record focal MAPs could be employed clinically to better assess a given lesion quality and/or to determine the existence of an undesired conduction gap.

## Graphical abstract

This paper describes a novel approach to examine unwanted conduction gaps in lesion sets following therapeutic ablation to treat atrial fibrillation. We demonstrated the use of a proof of concept device to identify conduction gaps in a linear lesion set. Furthermore, we were able to determine required contact forces to appropriately assess focal electrical changes of the underlying tissues.

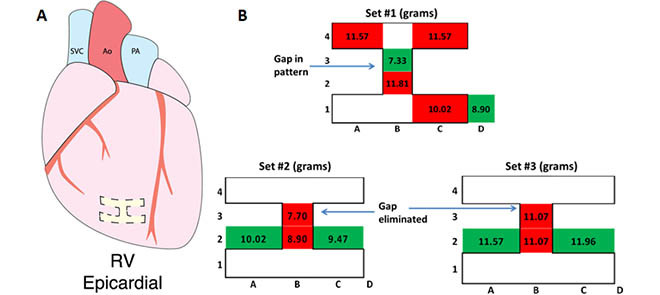

## I. Introduction

Post-ablation reoccurrences of atrial fibrillation (AF) continue to be a concern in the treatment of patients [1], [2]. Therefore, several adjuvant procedures have been promoted to improve overall outcomes. One of these, the ablation of the mitral isthmus (MI), has emerged as an attempt to decrease the likelihood of macro-reentrant tachycardias [3]–[6]. Nevertheless, the creation of a transmural MI block for the treatment of AF is often quite difficult to achieve, as appropriate catheter placement and sustained stability of tissue contact can be challenging due to a given patient’s anatomy [3]. For example, post-treatment incomplete lesion sets may in turn lead to slow conduction pathways that could result in left atrial tachycardia [7]–[10].

Today, in the cardiac catheterization laboratory, the creation of linear lesion sets of the MI is an employed method in many arrhythmia patients to decrease the likelihood of reoccurrence of macro-reentrant tachycardia [3], [4]. Importantly, the occurrence of post-ablative conduction gaps after such procedures has also been attributed to limitations of currently available catheter ablation devices [11]–[13]. In other words, the limited clinical ability to critically locate a potential conduction gap in a planned lesion presents an opportunity to develop innovative tools and/or techniques to improve the outcomes of these procedures. It should be noted that there have been previous attempts to design mapping/ablation catheters with the ability to specifically record monophasic action potentials (MAPs), but none remain in clinical practice today [14], [15]. In 2009, utilizing a porcine model, Aidonidis et al. [16] hypothesized that the patterns of local repolarization in the high and low right atrium would allow determination of the optimal sites with pronounced propensity for eliciting disorganization during atrial flutter and AF; to do so they used focal catheters that were capable of continuously recording MAPs. More specifically, they noted that by monitoring high atrial MAPs, they could identify discordant repolarizations during atrial flutter or AF, whereas the low atrial MAPs maintained their baseline regular morphologies. The primary objective of this technical brief was to evaluate the feasibility and potential clinical utility of simultaneously monitoring MAPs and contact forces (CFs) in a porcine animal model, as a means to improve the ability to locate conduction gaps in elicited linear radiofrequency (RF) lesions.

## II. Methodology

### A. Animal Model

**FIGURE 1.**
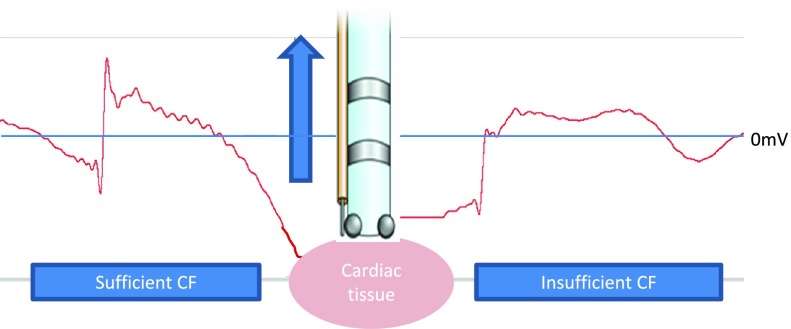
Catheter applied to the cardiac tissue with a transmembrane action potential moving past the tip of the catheter. Sufficient contact force (CF) of the catheter resulted in the ability to detect an underlying MAP. The catheter force was decreased, resulting in insufficient CF to record a MAP.

Swine hearts were reanimated with a clear Krebs-Henseleit buffer employing previously described Visible Heart methodologies, which also allowed us to record endoscopic and endocardial video footage during ablation procedures [17], [18]. These study protocols were reviewed and approved by the University of Minnesota Institutional Animal Care and Use Committee. Endocardial images using either a 4.0- or 6.0-mm diameter videoscope (Olympus, Center Valley, PA, USA) were obtained. MAP signals were characterized by determining relative occurrences and associated waveform properties relative to the tip location of a given catheter. Thus these signals enabled a more detailed anatomic/physiologic study of smaller areas of underlying myocardial substrate [16], [19]–[21]. MAP detection required achieving a threshold of activation as well as the application of proper contact forces (Fig. 1).

### B. Catheter Design

**FIGURE 2.**
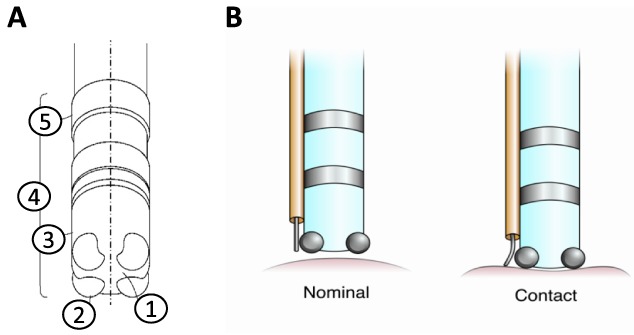
(A) Design of the tip of the MAP catheter (4) with 4 distal electrodes (2) and 2 2-mm band electrodes (5) that were mounted on the shaft of the catheter. The tip electrodes are isolated from each other using a polymer (1, 3); this catheter design is capable of recording both unipolar and bipolar signals. (B) Illustration of the catheter tip as it engages the myocardial or tissue surface. The electrodes and fiber Bragg grating strain sensor (orange tube) come in contact with the tissue at the same time. Contact is denoted by deformation of the fiber grating in the right panel of (B). Such a deformation was translated into grams of CF. CF=contact force, MAP=monophasic action potential.

A catheter was devised with the ability to simultaneously collect MAP signals and CFs (grams; Fig. 2); for the latter an optical fiber Bragg grating was utilized [22]. MAP signals were recorded as bipolar electrical potentials between catheter electrodes, using a filter bandwidth of 0.1 to 120 Hz for waveform collection. The appropriate collection of MAP signals was identified by the presence of a signal morphology of a hundred milliseconds or greater and a target amplitude more than 2 mV.

CFs were collected using a diffraction grating; the fiber Bragg grating was built into the optical fiber and reflected light at a specific wavelength. The activation of a given wavelength related to the strain applied to the fiber. An optical interrogator (HBM, Germany) was employed as a “swept wavelength” laser to determine the reflected wavelength, which was then utilized to determine the relative strain. Each catheter’s force sensor was constructed in short segments of optical fiber with an accuracy of ±1 nm. Strain was assumed with Bragg displacement in relation to a reference length, and was transformed into a normalized measure of deformation, i.e., the relative amount of strain that was generated was computed as a deformation. Each fiber Bragg grating strain sensor was calibrated before the start of the experimental protocol. To do so, the tip of the given fiber had a measureable force (grams) placed against it. The force was incrementally increased and frequency/force data were collected. This resulted in a frequency-to-force relationship that was used to derive a linear expression, i.e., the calibration curve. This calibration process enabled each unique piece of fiber to be normalized. Collection of ECG signals was performed using a bipolar electrogram with a filter bandwidth of 0.1 to 120 Hz. MAP data collection and subsequent analyses were performed using the ADInstruments Bioamp system (Dunedin, New Zealand).

### C. Catheter Ablation Assessment Protocol

**FIGURE 3.**
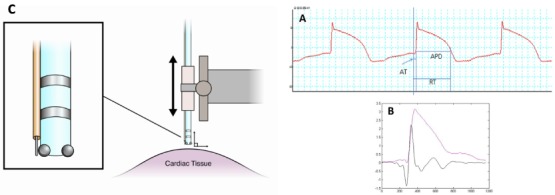
(A) Observable real-time signals that were also recorded for subsequent determinations of AT, APD, and RT. (B) A single MAP (purple) and ECG (black) signal simultaneously recorded from the RV epicardium (using both MAP and standard cardiac mapping catheters). (C) A stabilizing device with an incorporated micromanipulator was utilized for catheter orientations, aligning the directions of applied forces, and/or for maintaining catheter orientations for epicardial measurements. APD=action potential duration, AT=sum of activation time, ECG=electrocardiography, MAP=monophasic action potential, RT=repolarization time, RV=right ventricle.

This protocol ensured that prior to the ablation of tissue, ventricular MAP CF measurements of viable tissue were taken to determine the necessary CF to record a MAP. The endocardial and epicardial ablations were comprised of a series of applied linear lesions created with a focal RF ablation catheter. Direct visualization was used as a means to validate ablation anatomic locations as well as relative catheter positioning. We used comparative beat-to-beat measurements in cases of MAP distortions or any recognized disturbance to the baseline recording, e.g., due to cardiac contractions. Activation time (AT) was defined as the time interval from the earliest recorded ventricular activation to local activation; end-of-repolarization (EOR) time was the time interval from the earliest ventricular activation to local EOR; and MAP duration (MAPDUR) was determined as the time interval from local activation to local EOR (Fig. 3A). Relative parameters were obtained at each planned location utilizing the peaks of QRS complexes as references (Fig. 3B). To minimize relative signal variations associated with cardiac movements or other sources of interference, we measured the CF for a given catheter contact site after observing a series of 4 consecutive MAP waveforms that had similar characteristics. Prior to conducting the ablation, a series of epicardial ventricular septal CF measurements was taken on viable tissue. These measurements were collected to describe the amount of CF needed to generate a MAP.

To amplify and digitally record all MAP signals from each electrode on each catheter tip, we used a computer-based laboratory system (PowerLab, ADInstruments, Colorado Springs, CO, USA). Each MAP signal was recorded in a bipolar configuration, i.e., between tip electrodes and a 2-mm ring electrode. We also obtained a unipolar electrogram, with a filter bandwidth of 0.1 to 120 Hz. Increasing force was applied to a given application location until the first recognizable MAP signal was observed (Fig. 3). We used the CF at the time of the last set of 4 consecutive MAP signals to examine potential effects associated with observed tissue deformations, e.g., due to sustained forces from various catheter placements. Additionally for in situ data affected by respiration, we utilized a momentary breath hold for short periods of 10 to 20 seconds in order to reduce the impact of effects associated with lung movements relative to the heart.

In addition to collecting MAPs, we utilized 5 French bipolar mapping electrode catheters, either epicardially or endocardially, to simultaneously collect far-field electrical signals.

The process of determining the presence of a conduction gap included the collection of MAP signals pre-ablation and the required amount of CF on the tissue to do so. Tissue that elicited recognizable MAPs was termed *conductive*. Post ablation, all anatomic locations were reassessed to determine if a conduction gap existed. If the amount of CF applied to the location exceeded the pre-ablated CF and there was no MAP signal, the location was deemed *non-conductive*. If a MAP signal was present in an ablative location, it was termed a conductive *gap* in the linear lesion. The generation of a MAP resulting from catheter application served to indicate the point in time to determine the approximate amount of CF that was required in a given anatomical application, i.e., the moment a recognizable MAP was generated and the corresponding CF.

The epicardial lesion pattern that was used for these studies was fashioned after the work done by Ranjan et al. [11] who previously devised a method for the creation and assessment of a conduction gap for a linear lesion for epicardial application. We generated lesions employing a RF ablation system (Contactr catheter and Atakr RF generator, Medtronic, Inc., Minneapolis, MN, USA); an initial power setting of 30 W was used during the primary lesion creation. Additional applications of RF energies were used (50Wover 10-30 seconds) on regions within the lesion pattern where MAPs were present.

**FIGURE 4.**
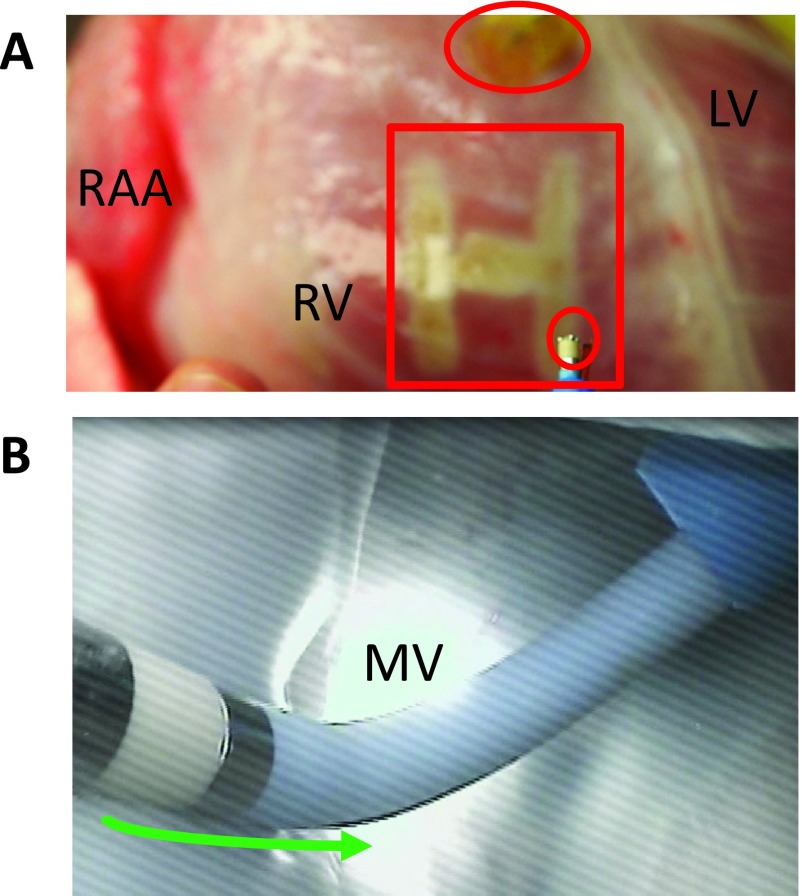
Surface view of a reanimated swine heart. (A) An epicardial linear lesion was generated on the right ventricle (RV) with the upper oval as an external pacing site and the lower oval containing the tip of a MAP catheter. (B) A left atrial endocardial image, showing the anatomical location of the mitral isthmus linear lesion, created by moving the ablation catheter towards the mitral valve annulus (green arrow). LV=left ventricle, MV=mitral valve, RAA=right atrial appendage.

To further assess the conduction properties of the myocardium, electrical stimuli were delivered to the right ventricular epicardium using constant current of 2-ms pulse duration at twice diastolic threshold, with a setting of 7-9 mA (Fig. 4A). Direct visualization also aided to confirm that the catheter electrodes and force sensors were located at the same anatomic location of a detectable lesion, to ensure accurate measurements were conducted (Fig. 4A, B).

In addition to the epicardially applied linear lesions, left atrial endocardial MI linear lesions were created, and again we subsequently assessed them for the presence of conduction gaps. CF data collection in both epicardial and endocardial locations was recorded in each measurement location once 4 consecutive MAP signals were observed. As before, this was done in part to compensate for the relative effects of cardiac movements, i.e., to prevent the premature identification of focal depolarizations. It should be noted that initial intermittent contact due to cardiac movement was more prevalent in epicardial assessments than the endocardial ones.

## III. Results

**FIGURE 5.**
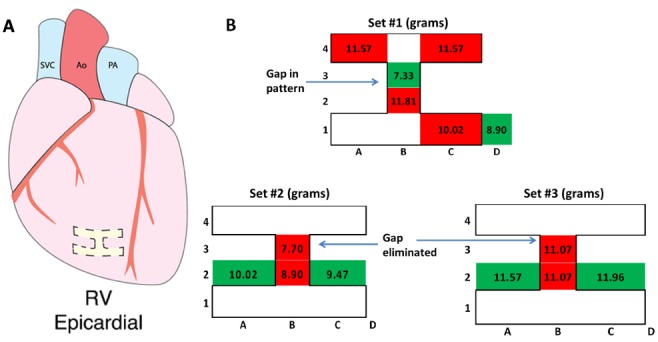
(A) An applied epicardial lesion pattern on the isolated reanimated swine heart on the right ventricle (RV). (B) The determined applied contact forces in different anatomic locations on or adjacent to the lesion pattern. Contact forces were indicated for each location site, with red indicating no detectable MAPs and green indicating viable tissue. Ao=aorta, PA=pulmonary artery, SVC=superior vena cava.

Prior to conducting ablations, >200 MAP points were collected at the tip of the catheter at the epicardial ventricular septum on 7 hearts with a mean CF of 7.6 ± 4.4 grams. Subsequently, the ablation pattern was created with several measurement points collected within the various anatomic ablation patterns on 1 heart. Shown in one pattern, Set #1 contained a conduction gap at location B3 (Fig. 5). After generation of the pattern, 6 CF measurements were taken from 5 locations within the lesion pattern and 1 outside the pattern, with MAP signals appearing at 2 locations (B3 and D1; green), with maximal CFs of 7.3 and 8.9 grams recorded respectively. Both of these locations were non-ablated myocardium (Fig. 5B).

No MAP signals were generated in the remaining 4 locations in this lesion pattern: A4, B2, C1, and C4 (red). These CFs also exceeded the mean CF from epicardial ventricular septum. A second ablation procedure was then conducted; the only ablation-related change was an additional application of RF in location B3.

MAP signal collection was attempted at each of the A1, B2, B3, and C2 sites, with no MAP detectable at locations B2 and B3 (red; Set #2). The CF measured at site B3 exceeded the mean CF to generate a MAP observed from the epicardial ventricular points and did not generate a MAP. A further validation of the lesion pattern was conducted by measuring for potential MAPs at locations A2, B2, and C2. In A2 and C2, which were locations outside the pattern, MAP signals were detected (green). Myocardial location B2 did not elicit a MAP signal even with a CF of 8.9 grams (red). The heart was then allowed to stabilize for a given time period, then Set #3 of potentials were collected; measurements were taken in the same locations as Set #2 without any additional RF ablations performed. The CFs were gradually increased at all location sites beyond the mean required CF, to generate a MAP to ensure that optimal contacts were applied in order to record MAPs. Location B3 was assessed again with CFs up to 11 grams and no MAPs were observable (red). A2 and C2 were outside the lesion pattern and both generated MAPs with appropriate force applications (peak force of 11.57 and 11.96 respectively). Like site B3, location B2 did not elicit MAPs even at elevated CFs (red).

In summary, this catheter approach was able to define the locations of the lesion gap in Set #1 and after an additional application of RF at the gap, the MAP signals were no longer elicited. Assessment validations were conducted by measuring MAPs at locations outside the lesion patterns, and each time MAP signals could be recorded. Finally, the application of increased CFs was applied to ensure more than adequate tissue contact; results showed an absence of MAPs at the original pattern gap location and the continued ability to collect MAPs outside the lesion pattern.

**FIGURE 6.**
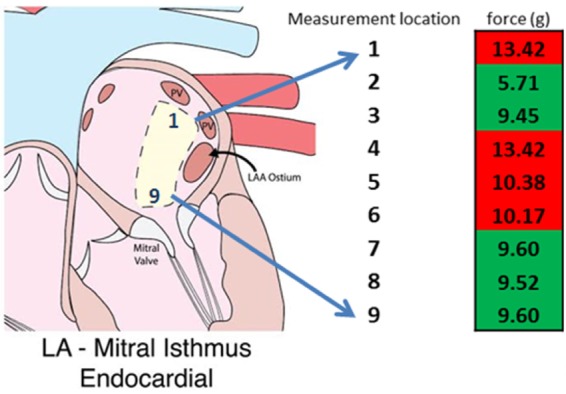
The recorded contact forces (grams) during which reproducible MAP signals could have been detected in 9 anatomical locations of the endocardium of the left atrial (LA) mitral isthmus. Red indicates that no MAPs were observed and green indicates that MAPs were recorded.

A similar ablation approach was used to assess the properties/integrity of endocardial left atrial MI ablations. Within a given specimen, a linear RF lesion was generated. The MAP catheter was then placed in 9 relative locations within the zones of the linear lesion, in an attempt to detect MAP waveforms and the associated CFs (Fig. 6).

Locations 1, 4, 5, and 6 did not result in the recording of MAPs (red), whereas the remaining locations did (green). In these latter instances, optimal MAP waveforms were observable at CFs less than 10 grams. Contact forces were increased to levels above 10 grams in locations where MAPs were considered not to be present.

## IV. Discussion

The inability to record MAPs within a created lesion zone, but yet with appropriately applied CFs, is a reproducible measure of an appropriate therapeutic application. The use of the mean CF of 7.6 grams serves as an indicator of the necessary force to record a reproducible MAP. Our sustained inability to record MAPs after ablation, at forces that exceed the mean CF of viable tissue, increases one’s confidence that tissues are no longer electrically viable. Yet, additional studies are needed to determine the necessary CFs required to record reproducible MAPs in various regions of the human myocardium. Further, if MAPs can be recorded from myocardial tissue in which there may be a conduction gap, this may help localize sites of additional ablations.

## V. Conclusion

Here we showed that use of an assessment catheter that allows for the detection of MAPs and CFs simultaneously has enhanced utility in assessing myocardial lesion quality. We utilized this approach to assess experimental linear lesions placed either endocardially or epicardially on functioning swine hearts; it relies on the effective application of a focal catheter.

Resulting lesions have varied sizes and depths relative to changes in tissue properties and/or changes in the amount of catheter contact during treatment applications. The use of an adjuvant method described here could help to acutely assess viability of myocardial tissue at the locations of the lesion at the time of the therapy. Thus, the use of a focal catheter which allows for the appropriate assessment of underlying MAPs may be a method to improve overall clinical ablation procedures, by providing an innovative way to understand changes in the myocardium at the time of ablative therapeutic applications.
